# Characteristics of serogroup 20 *S.pneumoniae* isolates from Brazil

**DOI:** 10.1186/s12879-016-1773-y

**Published:** 2016-08-15

**Authors:** Juliana Caierão, Fernando Hayashi Sant’Anna, Paulina Hawkins, Gabriela Rosa Cunha, Mariana Mott, Diego Rodrigues Falci, Pedro Alves d’Azevedo, Lesley McGee, Cícero Dias

**Affiliations:** 1 Basic Health Department, Federal University of Health Science of Porto Alegre, Porto Alegre, Brazil; 2 Genetic Departament, Bioscience Institute, Center of Agricultural Microbiology Federal University of Rio Grande do Sul, Porto Alegre, Brazil; 3 Rollins School of Public Health, Emory University, Atlanta, USA; 4 Conceição Hospital Group, Porto Alegre, Brazil; 5 Respiratory Diseases Branch, Centers for Disease Control and Prevention, Atlanta, USA

**Keywords:** *Streptococcus pneumoniae*, Invasive pneumococcal disease, Serogroup 20, Molecular epidemiology

## Abstract

**Background:**

Although serogroup 20 is not part of any conjugate pneumococcal vaccine, its serotype 20A, but not 20B, belongs to the polysaccharide 23-valent formula. Little is known about its clinical, laboratorial and epidemiological characteristics.

**Methods:**

The purpose of this study was to evaluate the bacterial genotypes (by PFGE and MLST), clinical characteristics of patients (from review of medical records) and antimicrobial susceptibility of serogroup 20 isolates which were recovered from patients with invasive pneumococcal disease (IPD) from 2007 to 2012. Subtyping to determine 20A and 20B types was also performed by sequencing the genes of the *cps* locus.

**Results:**

Sixteen isolates were genotyped and were highly related. All pneumococci were resistant to tetracycline and 31 % were non-susceptible to trimethoprim/sulfamethoxazole. Penicillin MIC ranged from 0.004 to 1 μg/mL and non-susceptibility (MIC ≥ 0.12 μg/mL) was observed in 5/16 isolates (31 %). All isolates belonged to subtype 20B. Most patients were male with a median age of 62 years and presented at least one underlying disease (mostly respiratory conditions). All isolates belonged to ST8889 and to a unique PFGE clone.

**Conclusions:**

A high clonal occurrence of serotype 20B pneumococci recovered from patients with IPD in Brazil was observed. As a non-PCV10 serotype, selective pressure may be responsible for this unusual occurrence of serogroup 20. However, temporal variation effect should not be underestimated; therefore it is an issue that warrants continued monitoring.

## Background

To reduce the burden of pneumococcal diseases, especially invasive cases, different vaccine formulations have been introduced worldwide [1–6].

Serogroup 20 is part of the 23-valent polysaccharide pneumococcal vaccine (PPV23), but it is not included in any of the available conjugate formulations. Albeit in a low proportion, this serotype has been found in the nasopharynx of children [7–12] and has also been reported as a cause of invasive disease [13–20]. Although little is known about specific characteristics related to its virulence, this serogroup has been associated with increased disease severity, invasiveness and mortality [15, 21]. In addition, serogroup 20 has also been linked with some clinically relevant resistance phenotypes, such as levofloxacin resistance [22].

In Brazil, serogroup 20 has recently been recognized among the more prevalent serotypes in the post-vaccine period [23]. Analysis of these isolates by multilocus sequence typing (MLST) identified them as belonging to the same (and newly described) sequence type (ST) 8889. Cálix and co-workers (2012) [24] subtyped isolates belonging to serogroup 20 based on differences in the *cps* locus and designated these variants as 20A and 20B. They showed distinguishable antigenicity when human sera from patients who were vaccinated with the PPV23 were used. The authors concluded that this vaccine contains serotype 20A polysaccharide, but not 20B.

Compared with other serotypes, there is a restricted number of strains of serogroup 20 described in the MLST database (http://pubmlst.org/spneumoniae/) and therefore additional molecular epidemiology analyses remain to be explored. The aim of this work was to characterize isolates of serogroup 20 recovered from patients with IPD in Porto Alegre, South Brazil.

## Methods

### Study setting and bacterial isolates

*S. pneumoniae* isolates from patients with IPD have been systematically collected as part of our surveillance studies. From January 2007 to December 2012, 358 pneumococci were recovered from patients attending three hospitals in Porto Alegre, Brazil: Hospital Mãe de Deus (HMD), Grupo Hospitalar Conceição (GHC) and Hospital de Clínicas de Porto Alegre (HCPA). One isolate per patient was considered. Identification was confirmed by optochin susceptibility and bile solubility tests [25]. Serotyping was performed for 336 isolates by multiplex PCR [26] and/or Quellung reaction. Among all *S. pneumoniae*, 4.8 % (16/336) were identified as serogroup 20 and included in the present study. Isolates were stored in Skim Milk® (Difco) with 5 % glycerol at -80 °C.

### Antimicrobial susceptibility tests

Minimal inhibitory concentration (MIC) to the following antimicrobials was determined by broth microdilution, as recommended by CLSI, 2013 [27]: penicillin, ceftriaxone, erythromycin, tetracycline, trimethoprim/sulfamethoxazole, levofloxacin, chloramphenicol and vancomycin. The reference strain *S. pneumoniae* ATCC 49619 was used for quality control.

### Genetic characterization of serogroup 20 cps locus

Regions of the capsular genes that previously demonstrated genetic variability between serotypes 20A and 20B [28] were sequenced: *wcjE*, *wchA* and *whaF*. DNA extraction was performed using a 5 % suspension of Chelex® 100 resin (Biorad). The oligonucleotide sequences used were designed based on the nucleotide sequence of the published serogroup 20 *cps* locus (GenBank™ accession number CR931679.1) and are shown in Table 1. PCR reaction included 1.2 μM of each primer, 2.5U of *Taq* DNA polymerase and 3.5 mM of MgCl_2_. Reaction conditions included an initial denaturation at 94 °C for 4 min, 30 cycles of denaturation (94 °C for 45 s), annealing (56 °C for 45 s) and extension (72 °C for 2.5 min) and a final extension at 72 °C for 5 min. PCR products were purified with ExoSAP-IT (Affymetrix USB, Santa Clara, CA) and cycle sequenced using the BigDye Terminator V3.1 chemistry (Life Technologies, Carlsbad, CA). Sequencing reactions were analyzed on an ABI 3130xl genetic analyzer (Applied Biosystems, Carlsbad, California, USA).

**Table 1:** Primers sequences used for PCR and sequencing

Region	Sequence	Amplicon (bp)	Reference
*wcjE*	5’-AGCCTTACTATCCGATCAACG-3’	1334	Calix et al, 2012 [24]
	5’-CTTGTTATGACGCGCTTACC-3’		Calix et al, 2012 [24]
*wchA*	5’-CCTGTTACTTGCGAACGATG-3’	374	Calix et al, 2012 [24]
	5’-GACCAACGATAGCTCCACAAA-3’		This article
*whaF*	5’- TGAATTTGAAGAGATAAGGGAAA-3’	457	This article
	5’-CCCGTGTTACATAAGGTGTTG-3’		This article

### Molecular typing

Pulsed-field gel electrophoresis (PFGE) and MLST techniques were used for molecular typing. PFGE was performed according to McEllistrem et al. (2000) [28] and Pinto et al. (2013) [29]. PFGE patterns were clustered by UPGMA. A dendrogram was generated from a similarity matrix calculated using the Dice similarity coefficient with an optimization of 0.5 % and a tolerance of 1.5 %. A non-invasive serogroup 20 isolate (079-12), recovered during the same period from a sputum sample, was included for comparative purposes. Clonal relationship among isolates was defined according to parameters published by Tenover and co-workers (1995) [30].

MLST was previously performed by our group [23], according to Enright & Spratt (1998) [31], using modified primers described at CDC’s Streptococcus Laboratory website (http://www.cdc.gov/streplab/alt-mlst-primers.html).

### Analysis of medical records

Patient’s records were evaluated to obtain information such as age, gender, intensive care unit (ICU) admission, outcome and occurrence of the following underlying conditions: diabetes, hypertension, HIV infection, liver diseases, chronic kidney failures, asplenia, chronic obstructive pulmonary disease, autoimmune disease, transplantation, neoplasia, smoking and alcoholism.

### Ethical considerations

This retrospective study was approved by the Research Ethical Committee of Grupo Hospitalar Conceição (Project number 11-205). Patient records and information were anonymized and de-identified prior to analysis.

## Results and discussion

From 2007 to 2012, 16 out of 336 pneumococci recovered from patients with IPD belonged to serogroup 20. Yildirim and co-workers (2012) [17] evaluated prevalence of serotypes causing invasive diseases in two periods: directly after PCV7 implementation in USA (2000-2002) and a few years later (2009-10) and also observed an increase in the proportion of invasive disease caused by serogroup 20. However, other studies have reported a stable participation of this serogroup in invasive disease over the years [15].

Yearly, the proportion of serogroup 20 increased from 2007 (1/43, 2 % were serotype 20) to 2011 (with 11/125, 9 % of all pneumococci serotyped as 20) and decreased after that (1/76, 1 % in 2012). Indeed, most of the cases of invasive disease caused by serogroup 20 were detected after the implementation of PCV10 (69 %; 11/16), mainly in the year 2011. However, such effect of the vaccine would be unlikely only one year after the beginning of the vaccination program, especially among adults, and in addition only one serogroup 20 case was observed in 2012. It is well known that temporal variations in the distribution of pneumococcal serotypes are expected, independent of selective pressure due to antibiotic use or vaccination and is a more likely explanation for the changes in prevalence seen in this study than is vaccine-related serotype replacement [32].

Most patients were male (56 %; 9/16). For two patients, no gender data were available. The age of patients varied from 37 to 85 years old and the average and median ages were 61.6 and 62 years old, respectively. Eight (50 %) patients were ≥65 years old. Most isolates were recovered from patients admitted at GHC (81 %; 13/16). Isolates from HCPA and HMD represented 12 % (2/16) and 6 % (1/16), respectively.

The majority of isolates (87 %; 14/16) were from blood and the remainder from cerebrospinal fluid (CSF). Although some authors have reported both invasive [13, 33] and non-invasive [20] infections caused by pneumococci from serogroup 20 in adults, studies consistently describe this serogroup in invasive disease among children [15, 17, 19, 36]. Most studies report serogroup 20 associated with bacteremia and/or meningitis [21, 34]. Also, serogroup 20 appears to be found in a very small proportion of pneumococci in the nasopharynx of children [7–12].

Medical records were available for 12 patients (Table 2). All of them presented at least one underlying condition. The most common underlying conditions were chronic obstructive pulmonary disease (COPD) (4/12; 33 %), alcoholism (4/12; 33 %), systemic arterial hypertension (5/12; 42 %) and smoking (5/12; 42 %). Indeed, it is well-established that pneumococcal diseases (considering all serotypes) are facilitated by abnormal conditions of the respiratory tract or underlying conditions [6]. Three patients (25 %) needed admission to ICU.

**Table 2:** Clinical manifestations of patients presenting with invasive disease caused by serogroup 20 *S. pneumoniae* isolates

ID#	Date^a^	Origin	Source	Age	DM	SAH	HIV	Liver disease	COPD	Neoplasia	Smoking	Alcoholism	ICU	Death
009–07	pre	GHC	blood	NA	NA	NA	NA	NA	NA	NA	NA	NA	NA	NA
044–09	pre	GHC	blood	NA	NA	NA	NA	NA	NA	NA	NA	NA	NA	NA
045–09	pre	GHC	blood	85–89	no	yes	no	no	no	no	no	no	no	no
079–09	pre	GHC	CSF	55–59	no	yes	no	no	yes	no	yes	yes	no	no
014–11	pre	GHC	blood	70–74	yes	no	no	no	no	yes	no	no	no	no
056–11	post	GHC	blood	50–54	no	no	yes	no	no	no	no	no	no	no
089–11	post	HCPA	blood	NA	NA	NA	NA	NA	NA	NA	NA	NA	NA	NA
103–11	post	HCPA	CSF	NA	NA	NA	NA	NA	NA	NA	NA	NA	NA	NA
114–11	post	GHC	blood	70–74	yes	yes	no	no	yes	no	yes	no	no	no
121–11	post	GHC	blood	65–69	no	yes	no	no	yes	no	No	no	no	no
124–11	post	GHC	blood	65–69	no	no	no	yes	yes	no	yes	yes	yes	yes
130–11	post	GHC	blood	35–39	no	no	no	no	no	no	yes	yes	yes	no
136–11	post	GHC	blood	65–69	no	no	no	no	no	yes	yes	no	no	no
142–11	post	GHC	blood	75–79	no	no	no	no	no	yes	no	no	no	yes
161–11	post	HMD	blood	50–54	no	no	no	no	no	no	no	no	no	no
031–12	post	GHC	blood	80–84	yes	yes	no	no	no	no	no	no	no	no

^a^Date is referred to periods pre-vaccination (from 2007 to 2010) and post-vaccination (from 2010 to 2012)

*GHC* Grupo Hospitalar Conceição, *HCPA* Hospital de Clínicas de Porto Alegre, *HMD* Hospital Mãe de Deus, *NA* not available, *DM* diabetes mellitus, *SAH* systemic arterial hypertension, *COPD* chronic obstructive pulmonary disease, *ICU* admission to intensive care unit

Five of the 12 (42 %) patients with known outcome died. However, it is difficult to established mortality attributable to pneumococcal infection as underlying conditions were a common feature. Despite these confounders, some studies have demonstrated an increased risk of invasive disease and/or poor outcome related to serogroup 20. In this context, Grabenstein and co-workers (2014) [33] performed a systematic review to characterize differences in serious outcome between pneumococcal serotypes; among seven adult studies evaluating meningitis, serogroup 20 was among the group with elevated risk. Also, Jansen and co-workers (2009) [13] demonstrated that serogroup 20 was among the serotypes that caused meningitis and bacteremia without focus in a relatively high proportion compared to other serotypes. Other studies focusing on analysis of invasiveness demonstrate that this serogroup, along with others, was found to have an enhanced propensity to cause invasive disease [21]. Despite this important characteristic of invasiveness, serogroup 20 was associated with low rates of case-fatality [13]. In contrast, eight deaths among 569 IPD cases reported by Hsu and co-workers (2010) [15] were associated with a group of non-PCV7 isolates, including serogroup 20.

All pneumococci were resistant to tetracycline and 31 % (5/16) were non-susceptible to trimethoprim/sulfamethoxazole (2 with intermediate resistance and 3 fully resistant). Interestingly, we had previously characterized the antimicrobial susceptibility profile of 159 pneumococci recovered from invasive disease in our region [35] and resistance to tetracycline was observed in 22 % of the isolates. Penicillin MICs ranged from 0.004 to 1 μg/mL and resistance (MIC ≥ 0.12 μg/mL) was observed in 31 % (5/16) MIC_50_ for penicillin was very low (<0.03 μg/mL); MIC_90_ was 0.5 μg/mL and one isolate had an MIC of 1.0 μg/mL. Among the limited number of available studies describing antimicrobial susceptibility profiles of serogroup 20 isolates, non-susceptibility to penicillin was not observed by Dunais et al (2011) [7], while one paper reported MICs higher than 0.12 μg/mL [36]. Our isolates were susceptible to all other antimicrobials tested. Indeed, as described in literature, resistance to other antimicrobials appears to be low. Rudolph and co-workers (2013) [37] found a very small proportion of isolates of serogroup 20 non-susceptible to erythromycin among invasive pneumococci recovered from Alaska (1986–2010). Recently, Guo and co-workers (2014) [22] reported two pneumococci belonging to serotype 20B presenting resistance to quinolones in China.

**Fig. 1:**
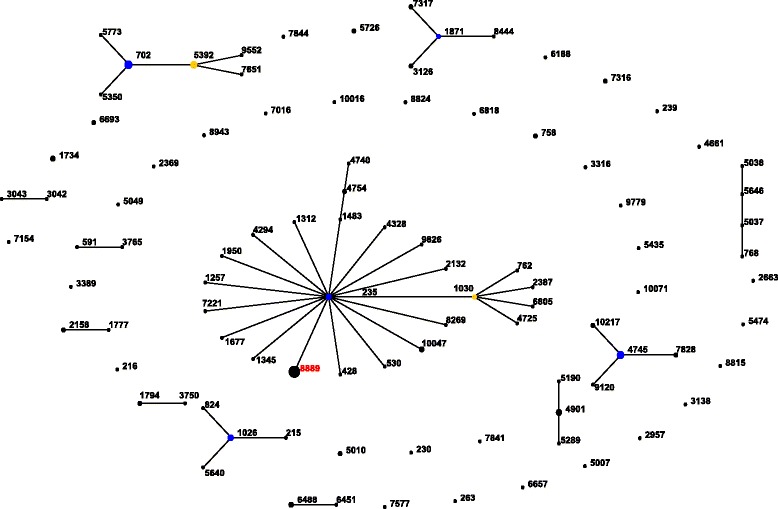
Population snapshot of 151 serogroup 20 isolates in the *S.pneumoniae* MLST database (accessed in April 2015) based on eBURST analysis. Each ST encountered is indicated by a circle, with their diameters being proportional to the numbers of isolates. ST described by our group [23] is highlighted in red

All but one isolate (121–11) were previously submitted to the MLST website; all isolates represented a unique and newly described sequence type: ST8889 [23]. Rudolph and co-workers (2013) [37] typed an erythromycin non-susceptible serogroup 20 isolate and found it to be ST1030, which is one of the 95 serogroup 20 sequence types listed at the MLST website. These STs are distributed in 12 groups (with 59 STs) and 35 singletons. ST 8889 is part of the major clonal complex (CC235) and is a single locus variant of the ancestor, ST235 (Fig. 1).

**Fig. 2:**
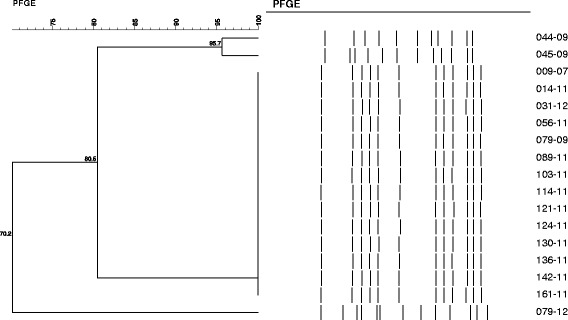
Representative dendogram of the invasive serogroup 20 pneumococci generated by PFGE. A non-invasive isolate belonging to serogroup 20 (079-12) was included for molecular epidemiology comparison, only

PFGE grouped all the 16 invasive isolates in a single clone (similarity of 80 % or more in the band pattern). Most isolates (87 %; 14/16) had an identical band profile, and the remaining two pneumococci (044–99 and 045–99) presented a very similar band pattern (Fig. 2). The two isolates recovered from CSF were included in the major PFGE band profile and the ones recovered from patients attending different hospitals (HMD and HCPA) also presented the major band profile. Interestingly, the non-invasive isolate (079–12) included for comparison purposes presented the most distinct band pattern, as shown in Fig. 1, and it was not included in the same clone.

According to Calix and co-workers (2012) [24], serotype 20B (GenBank™ accession number JQ653093.1) is identical to the reference strain of serogroup 20 (GenBank™ accession number CR931679.1), except for the occurrence of a silent mutation in *wcjE* (936 c::t). On the other hand, serotype 20A (GenBank™ accession number JQ653094.1) presents three alterations in genes of the *cps* locus: mutation in *wchA* (659 t::g), mutation in *whaF* (898 a::c) and an adenosine insertion within a polyadenosine tract in *whaF* (position 881). None of the 16 pneumococci had these alterations. The *wchaA* gene of our isolates was identical to the strains CR931679.1 and JQ653093.1 (20B), as well as for *whaF*. The *wcjE* gene of isolates included in this study did not have the 936 c::t mutation observed in 20B (JQ653093.1). However, as this is a silent mutation, we can deduce that the protein constitution of our isolates is the same as the previously described 20B. Serotype 20B seems to be the more common subtype identified amongst serogroup 20 isolates, at least among the restricted number of serogroup 20 isolates subtyped so far. Our subtyping results correlated with Calix et al (2012) [24] who identified serotype 20B amongst their isolates, which were also all from IPD.PPV23 includes serotype 20A and although Calix and co-workers [24] infer there might be effective cross-protection against 20B, they suggest epidemiological analyses are warranted to confirm these data.

## Conclusions

Serogroup 20 is an infrequent non-PCV serotype and little is known about its molecular epidemiology and clinical disease presentation. As changes in the pneumococcal population are expected due to temporal variation and/or selective pressure of vaccination/antimicrobial use, it is important to generate data to better understand the evolution of serotypes not included in available conjugate vaccine formulations. As far as we know, this is the first study devoted to this serotype in Latin America. We focused on the occurrence of a specific ST of serotype 20B, a serotype not included neither in the conjugate nor polysaccharide vaccines. This observation leads to the need of further surveillance for this non-vaccine serotype.

## Abbreviations

GHC: Grupo Hospitalar Conceição
HCPA: Hospital de Clínicas de Porto Alegre
HMD: Hospital Mãe de Deus
ICU: Intensive care units
IPD: Invasive pneumococcal diseases
MIC: Minimal inhibitory concentration
MLS: Multilocus sequence typing
PCR: Polymerase chain reaction
PCV: Pneumococcal conjugate vaccine
PFGE: Pulsed-field gel electrophoresis
PPV23: Pneumococcal polyssacharyde vaccine
ST: Sequence type.

## References

[1] Welte T, Torres A, Nathwani D (2012). Clinical and economic burden of community-acquired pneumonia among adults in Europe. Thorax.

[2] Harboe ZB, Valentiner-Branth P, Benfield TL, Christensen JJ, Andersen PH (2010). Early effectiveness of heptavalent conjugate pneumococcal vaccination on invasive pneumococcal disease after the introduction in the Danish Childhood Immunization Programme. Vaccine.

[3] Pilishvili T, Lexau C, Farley MM, Hadler J, Harrison LH (2010). Sustained reductions in invasive pneumococcal disease in the era of conjugate vaccine. J Infect Dis..

[4] Afonso ET, Minamisava R, Bierrenbach AL, Escalante JJC, Alencar AP (2013). Effect of 10-Valent pneumococcal vaccine on pneumonia among children, Brazil. Emerg Infect Dis.

[5] Miller E, Andrews NJ, Waight PA, Slack MP, George RC (2011). Herd immunity and serotype replacement 4 years after seven-valent pneumococcal conjugate vaccination in England and Wales: an observational cohort study. Lancet Infect Dis..

[6] Grau I, Ardanuy C, Calatayud L, Rolo D, Domenech A, Liñares J, Pallares R (2012). Invasive pneumococcal disease in healthy adults: increase of empyema associated with tnhe clonal-type Sweden(1)-ST306. PLoS One.

[7] Dunais B, Bruno-Bazureault P, Carsenti-Dellamonica H, Touboul P, Pradier C (2011). A decade-long surveillance of nasopharyngeal colonization with *Streptococcus pneumoniae* among children attending day-care centers in south-eastern France: 1999-2008. Eur J Clin Microbiol Infect Dis..

[8] Ercibengoa M, Arostegi N, Marimón JM, Alonso M, Pérez-Trallero E (2012). Dynamics of pneumococcal nasopharyngeal carriage in healthy children attending a day care center in northern Spain. Influence of detection techniques on the results. BMC Infect Dis.

[9] Scott JR, Millar EV, Lipstich M, Moulton LH, Weatherholtz R, Perilla MJ, Jackson DM, Beall B, Craig MJ, Reid R, Santossham M, O´Brien KL (2012). Impact of more than a decade of pneumococcal conjugate vaccine use on carriage and invasive potential in Native American communities. J Infect Dis.

[10] Ansaldi F, de Florentiis D, Canepa P, Zancolli M, Martini M, Orsi A, Durando P, Icardi G (2012). Carriage of *Streptococcus pneumoniae* years after implementation of vaccination program in a population with very high and long-lasting coverage, Italy. Vaccine.

[11] Dashti A, Abdinia B, Karimi A (2012). Nasopharyngeal carrier rate of *Streptococcus pneumoniae* in children: serotype distribution and antimicrobial resistance. Arch Iran Med..

[12] Tigoi CC, Gatakaa H, Karani A, Mugo D, Kungu S, Wanjiru E, Jomo J, Musyimi R, Ojal J, Glass NE, Abdullahi O, Scott JA (2012). Rates of acquisition of pneumococcal colonization and transmission probabilities by serotype, among newborn infants in Kilifi District, Kenya. Clin Infectr Dis.

[13] Jansen AGSC, Rodenurg GR, van der Ende A, van Alphen L, Veenhoven RH (2009). Invasive pneumococcal disease among adults: association among serotypes, disease characteristics and outcome. Clin Infect Dis..

[14] Melegaro A, Choi YH, George R, Edmunds WJ, Miller E, Gay NJ (2010). Dynamic models of pneumococcal carriage and the impact of the heptavalent pneumococcal conjugate vaccine on invasive pneumococcal disease. BMC Infect Dis..

[15] Hsu KK, Shea KM, Stevenson AE, Pelton SI (2010). Massachusetts Department of Public Health. Changing serotypes causing childhood invasive pneumococcal disease: Massachusetts, 2001-2007. Pediatr Infect Dis J.

[16] Menezes AP, Campos LC, dos Santos MS, Azevedo J, dos Santos RC (2011). Serotype distribution and antimicrobial resistance of *Streptococcus pneumoniae* prior to introduction of the 10-valent pneumococcal conjugate vaccine in Brazil, 2000-2007. Vaccine.

[17] Yildirim I, Stevenson A, Hsu KK, Pelton SI (2012). Evolving Picture of invasive pneumococcal disease in Massachusetts children: a comparison of disease in 2007-2009 with earlier periods. Pediatr Infect Dis J..

[18] Donkor ES, Davie NT, Badoe EV (2013). Vaccination against pneumococcus in West Africa: perspectives and prospects. Int J Gen Med..

[19] Liu C, Xiong X, Xu W, Sun J, Wang L, Si J (2013). Serotype and patterns of antibiotic resistance in strains causing invasive pneumococcal disease in children less than 5 years of age. PLoS One.

[20] Mayanskiy N, Alyabieva N, Ponomarenko O, Lazareva A, Katosova L, Ivanenko A, Kulichenko T, Namazova-Baranova L, Baranov A (2014). Serotype and antibiotic resistance of non-invasive *Streptococcus pneumoniae* circulating in pediatric hospitals in Moscow, Russia. Int J Infect Dis.

[21] Sá-Leão R, Pinto F, Aguiar S, Nunes S, Carriço JA, Frazão N, Gonçalves –Souza N, Melo-Cristino J, de Lancastre H, Ramirez M (2011). Analysis of invasiveness of pneumococca serotypes and clones circulating in Portugal before widespread use of conjugate vaccines reveals heterogeneous behavior of clones expressing the same serotype. J Clin Microbiol.

[22] Guo Q, Zhuo C, Xu Y, Huang W, Wang C, Zhang S, Huang J, Hu F, Zhu D, Yang F, Wang M (2014). Genetic diversity of fluorquinolone-nonsusceptible *Streptococcus pneumoniae* clinical isolates and the first identification of serotype 20B in China. Eur J Clin Microbiol Infect Dis..

[23] Caierão J, Haekins P, Sant’anna FH, da Cunha GR, d’Azevedo PA, McGee L, Dias C (2014). Serotype and genotype inf invasive *Streptococcus pneumoniae* before and after PCV10 implementation in Southern Brazil. PLoS One.

[24] Calix JJ, Porambo RJ, Brady AM, Larson TR, Yother J (2012). Biochemical, genetic, and serological characterization of two capsule subtypes among *Streptococcus pneumoniae* serotype 20 strains: discovery of a new pneumococcal serotype. J Biol Chem..

[25] Spellerberg B, Brandt C, Versalovic J, Carroll KC, Funke G, Jorgensen JH, Landry ML, Warnock DW (2011). Streptococcus. Manual of Clinical Microbiology.

[26] Dias CA, Teixeira LM, Carvalho MG, Beall B (2011). Sequential multiplex PCR for determining capsular serotypes of pneumococci recovered from Brazilian children. J Med Microbiol..

[27] CLSI (2013). Performance Standards for Antimicrobial Susceptibility testing. Twenty-third informational Supplement. M100-S23.

[28] McEllistrem MC, Stout JE, Harrison LH (2000). Simplified protocol for pulsed-field gel electrophoresis analysis of *Streptococcus pneumoniae*. J Clin Microbiol..

[29] Pinto TC, Souza AR, de Pina SE, Costa NS, Borges Neto AA, Neves FP, Merquior VL, Dias CA, Peralta JM, Teixeira LM (2013). Phenotypic and molecular characterization of optochin-resistant *Streptococcus pneumoniae* isolates from Brazil, with description of five novel mutations in the ATPC gene. J Clin Microbiol..

[30] Tenover FC, Arbeit RD, Georing RV, Mickelsen PA, Murray BE, Persing DH, Swaminathan B (1995). Interpreting chromosomal DNA restriction patterns produced by pulsed-field gel electrophoresis: criteria for bacterial strain typing. J Clin Microbiol..

[31] Enright MC, Spratt G (1998). A multilocus sequence typing scheme for *Streptococcus pneumoniae*: identification of clones associated with serious invasive disease. Microbiology..

[32] Andam CP, Hanage WP. Mechanisms of genome evolution of *Streptococcus* Mechanisms of genome evolution of *Streptococcus*. Infect Genet Evol. 2014 doi:10.1371/journal.pone.0111129 10.1016/j.meegid.2014.11.007 PMC4430445 25461843

[33] Grabenstein JD, Weber DJ (2014). Pneumococcal serotype diversity among adults in various countries, influenced by pediatric pneumococcal vaccination uptake. Clin Infect Dis..

[34] Saha SK, Naheed A, El Arifeen S, Islam M, Al-Emran H, Amin R, Fatima K, Brooks WA, Breinman RF, Sack DA, Luby SP (2009). Pneumococcal Study Group. Surveillance for invasive *Streptococcus pneumoniae* disease among hospitalized children in Bangladesh: antimicrobial susceptibility and serotype distribution. Clin Infect Dis.

[35] Mott M, Caierão J, Rosa da Cunha G, Rodrigues Perez LR, Matusiak R, de Oliveira KR P, d’Azevedo PA, Dias C (2014). Susceptibility profiles and correlation with pneumococcal serotypes soon after implementation of the 10-valent pneumococcal conjugate vaccine in Brazil. Int J Infect Dis.

[36] Sousa NG, Sá-Leão R, Crisostomo MI, Simas C, Nunes S, Frazão N, Carriço JÁ, Mato R, Santos-Sanches I, de Lancastre H (2005). Properties of novel international drug-resistant pneumococcal clones identificed in Day-care centers of Lisbon, Portugal. J Clin Microbiol.

[37] Rudolph K, Bulkow L, Bruce M, Zulz T, Reasonover A, Harker-Jones M, Hurlburt D, Hennessy T (2013). Molecular resistance mechanisms of macrolide-resistant invasive *Streptococcus pneumoniae* isolates from Alaska, 1986 to 2010. Antimicrob Agents Chemother..

